# Application of Genetic Algorithm to Hexagon-Based Motion Estimation

**DOI:** 10.1155/2014/689294

**Published:** 2014-02-02

**Authors:** Chih-Ming Kung, Wan-Shu Cheng, Jyh-Horng Jeng

**Affiliations:** ^1^Department of Information Technology & Communication, Shih Chien University, No. 200 University Road, Neimen District, Kaohsiung City 84550, Taiwan; ^2^Institute of Computer Science & Information Engineering, National Cheng Kung University, No. 1 University Road, Tainan City 70101, Taiwan; ^3^Department of Information Engineering, I-Shou University, No. 1 Section 1, Syuecheng Road, Dashu District, Kaohsiung City 84001, Taiwan

## Abstract

With the improvement of science and technology, the development of the network, and the exploitation of the HDTV, the demands of audio and video become more and more important. Depending on the video coding technology would be the solution for achieving these requirements. Motion estimation, which removes the redundancy in video frames, plays an important role in the video coding. Therefore, many experts devote themselves to the issues. The existing fast algorithms rely on the assumption that the matching error decreases monotonically as the searched point moves closer to the global optimum. However, genetic algorithm is not fundamentally limited to this restriction. The character would help the proposed scheme to search the mean square error closer to the algorithm of full search than those fast algorithms. The aim of this paper is to propose a new technique which focuses on combing the hexagon-based search algorithm, which is faster than diamond search, and genetic algorithm. Experiments are performed to demonstrate the encoding speed and accuracy of hexagon-based search pattern method and proposed method.

## 1. Introduction

**Figure 1:**
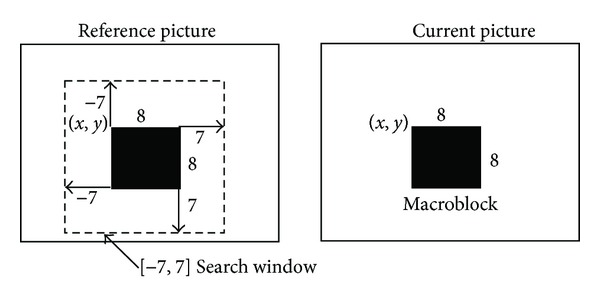
The reference picture with search window.

With the high compression ratio requiring and the channel bandwidth limiting, video coding is an indispensable process for many visual communication applications. In a video, sequence between successive frames which would have a great amount of similarity called temporal redundancy requires properly identifying and elimination to achieve coding the difference between frames. Block matching motion estimation which aimed to reduce such strong temporal redundancy between successive frames has been adopted by numerous video-coding standards. After parting a current frame into nonoverlaping equal size blocks, block matching distortion algorithms attempt to find the best-matched block in the current frame from a reference frame. In the reference picture, the block moves inside a search window that is centered on the position of the block in the current frame as depicted in Figure 1. The matching criterion of mean square error (MSE) would be used for finding the best case between the pair of blocks.

**Figure 2:**
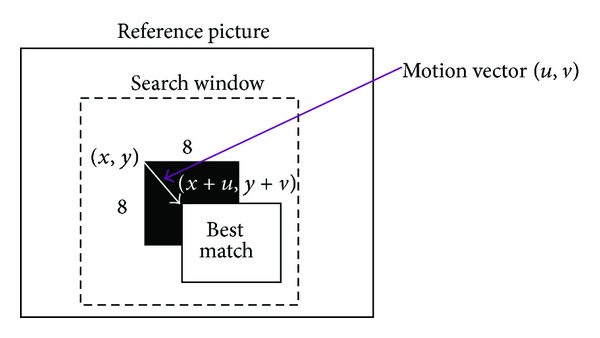
The best-matched reference block composes the motion vector.

The motion vector would be obtained from the best-matched reference block as shown in Figure 2. Full search algorithm is the most direct method, which provides the optimal result after matching all possible candidates within the search window. It is just because full search exhaustively evaluates all possible candidate blocks within the search window, the computation of the method is very intensive. But the time cost is very high and the computation is complicated. Therefore, fast and accurate block-based search technique is highly desirable to reduce processing delay while maintaining good reconstructed image quality. Many other popular fast block matching algorithms reduce computational complexity by limiting the number of checking points within the search window. Typical methods are developed seriatim, such as three-step search (TSS) [1], four-step search (4SS) [2, 3], diamond search (DS) [4], and hexagon-based search (HEXBS) [5]. In TSS, NTSS, and 4SS algorithms, square-shaped search patterns of different sizes are employed. On the other hand, the DS algorithm adopts a diamond-shaped search pattern, which has demonstrated faster processing with similar distortion in comparison with TSS, NTSS, and 4SS. After an in-depth examination, the influence of search pattern compared to diamond search pattern on speed performance, Zhu et al. [5] proposed a novel algorithm using a hexagon-based search pattern. And then, Zhu et al. demonstrate the method significant speedup gain over the diamond-based search. Although diamond search pattern speeds up the performance and reduces the searching points, the obtained motion vector after searching algorithm is not exactly the truly optimal solution.

In [6], an evolutionary control paradigm for vision-system pose planning for object motion estimation is proposed. A hybrid genetic algorithm is proposed to search for the optimal vision system pose that is occlusion free. Saha et al. [7] present a boundary-based approach towards pixel decimation with applications in block-matching algorithms (BMAs). Apart from the boundary-based approach, the novelty in our contribution also lies in performing a genetic algorithm- (GA-) based search to find optimal *M*-length patterns in an *N* × *N* block. Tagliasacchi illustrates a new optical flow estimation technique that builds upon a genetic algorithm (GA) [8]. For each region, a two-parameter motion model is estimated using a GA. The fittest individuals identified at the end of this step are used to initialize the population of the second step of the algorithm, which estimates a six-parameter affine motion model, again using a GA. The proposed method is compared with a multiresolution version of the well-known Lucas-Kanade differential algorithm. For promoting the performance of the speed and distortion, the proposed method tries to get rid of the limiting which is the assumption that distortion measurement decreases monotonically as the search point gets closer to the global minimum [9–12].

In this paper, we propose genetic algorithm methods to improve the performance of hexagon-based search method. In the following, diamond search pattern, hexagon-based search pattern, and genetic algorithm will be introduced in detail.

## 2. Fast Block Motion Estimation Algorithm

It is obvious that, in using block motion algorithm, the most accurate strategy is the full search (FS) method which exhaustively evaluates all possible candidate motion vectors over a predetermined neighborhood search window to find the optimum. The candidate that gives the best match for a given block distortion measure is chosen as the estimated motion vector. Nevertheless, this method has not been a popular choice because the high computational cost loading is needed. To overcome the drawback that reduced number of search points for finding the optimum motion vector, many fast block matching algorithms have exploited different search pattern and search strategies.

### 2.1. Diamond Search Pattern

**Figure 3:**
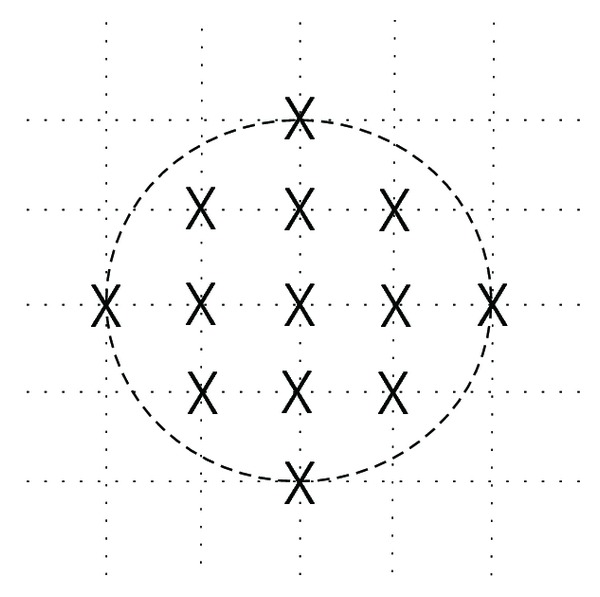
An appropriate search pattern is a circular area with a radius of two pixels.

A fast motion estimation algorithm named diamond search (DS) was first introduced in [4] and was proposed to promote the performance, which decrease the number of block matching within the search window. The search pattern's shape and size exploited in the fast algorithm have an important influence on not only its search speed but also distortion performance. The error surface is usually not monotonic especially for those image sequences with large motion content. Generally, multiple local minimum points exist in the search window. For example, the one used in BBGDS [13] with size of 3 × 3 searching with a small search pattern is quite likely to be trapped into a local minimum for those video sequences with large motion content. On the other hand, a large search pattern with size of 9 × 9 and fewer checking points as exploited in the first step of TSS is most likely to mislead the search path to a wrong direction and hence miss the optimum point. The DS pattern can find large motion blocks with fewer search points and also reduce to get stuck susceptibility in local optimum due to its relatively large step size in horizontal and vertical directions. The compact shape of the DS pattern around the center also yields fewer search points than 4SS for finding stationary or small motion vector. Square-shaped search patterns of different size are employed in TSS, NTSS, and 4SS algorithms. However, the DS algorithm uses a diamond-shaped search pattern, which has demonstrated faster processing with similar distortion in comparison with those techniques mentioned above. The motion vector of the block displacement of real-world video sequences could be in any direction, but mainly in horizontal and vertical directions. Based on these two crucial observations, the search points incurred within the circle with radius of two pixels are the most appropriate ones to be chosen to compose the search pattern as illustrated in Figure 3.

**Figure 4:**
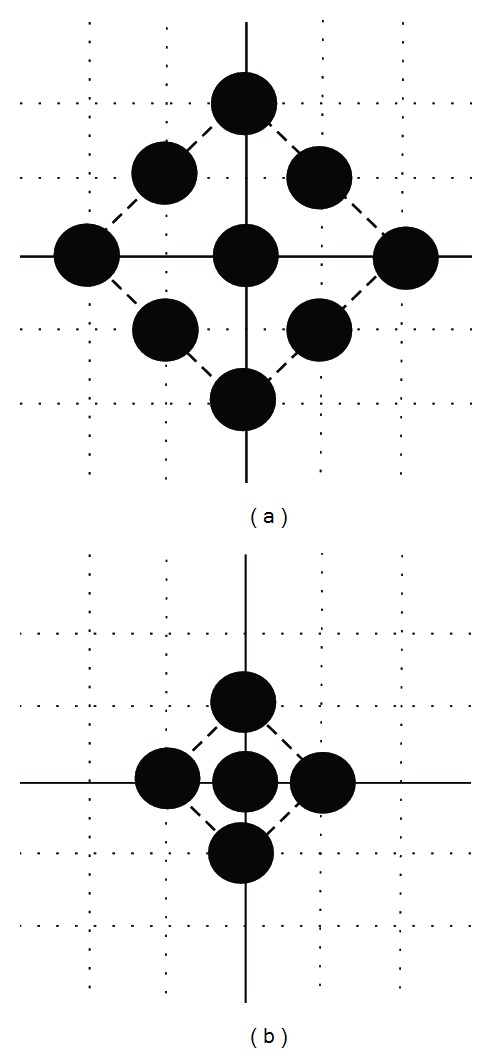
(a) Large diamond search pattern (LDSP). (b) Small diamond search pattern (SDSP).

The DS algorithm employs two basic search patterns as shown in Figure 4, which are derived from the crosses in Figure 3. The large diamond search pattern (LDSP) comprises the MSE of the nine checking points which are formed by the eight points surround the center one to compose a diamond shape as shown in Figure 4(a). The same searching procedure repeats until the block with the minimum MSE just locates on the center of diamond and again finds the minimum MSE using the SDSP as illustrated in Figure 4(b).

**Figure 5:**
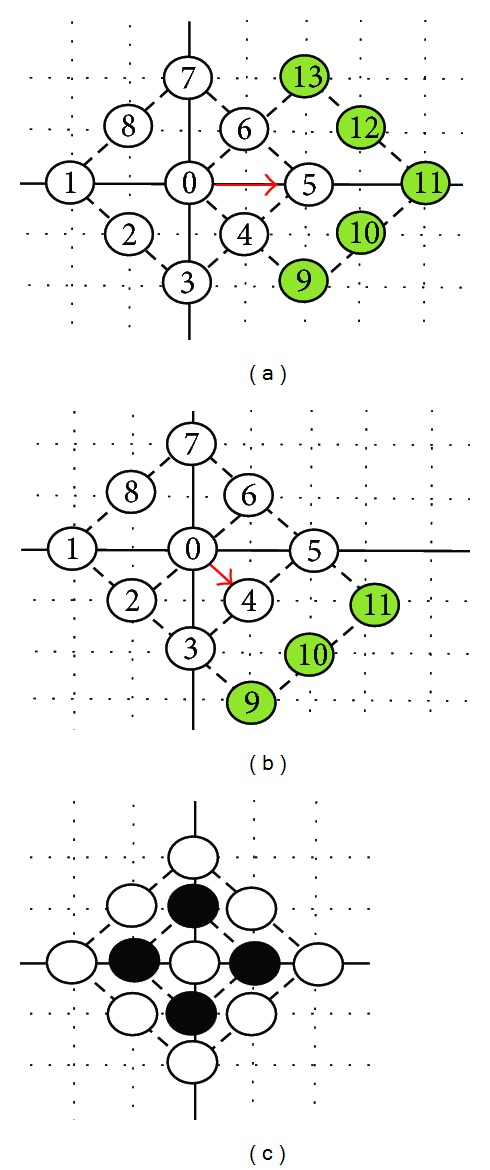
(a) The corner point. (b) DS along the edge point. (c) The minimum in the center point.

The checking points are partially overlapped between adjacent steps when LDSP is repeatedly used. For illustration in Figure 5, three cases of checking point overlapping are presented. When the previous minimum block distortion point is located at one of the corners or edge point of LDSP, only five or three new checking points are required to be calculated as shown in Figures 5(a) and 5(b), respectively. If the center point of LDSP produces the minimum block distortion, the final search stage would be used. In the final search, the search pattern would be switched from LDSP to SDSP. The small diamond search pattern (SDSP) as depicted in Figure 5(c) consisting of five checking points forms a smaller diamond shape. Among the five checking points in SDSP, the best matching block would be obtained for motion vector.

**Figure 6:**
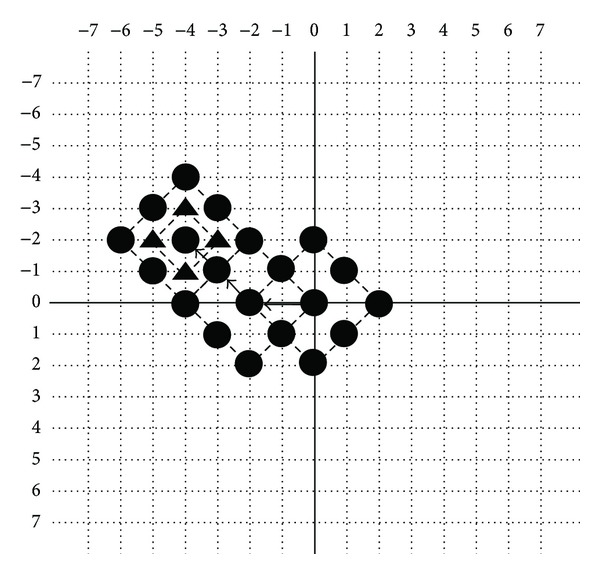
Search path example.

The instance shown in Figure 6 would be understood as the overlapping procedure when checking points with possible searching path using DS algorithm within a 15 × 15 search window. The starting search pattern will be formed by eight points which centered the position (0,0). After getting the minimum MSE position (−2, 0), next step will produce five new points which centered the position (−2, 0) to form another large diamond search pattern. In the next calculating, when (−3, −1) is the minimum block distortion, the next large diamond search pattern will be formed by producing three new points centered by the point (−3, −1) for next calculating. The algorithm will keep the same large diamond search pattern, until the minimum block distortion locates on the center position. Then, the small diamond search pattern will be used for the final deciding points for motion vector.

The diamond search pattern algorithm is summarized as follows:

**Step 1**

The original LDSP is centered at (0,0) of the search window and then checking the nine points for finding the best match. If the best match occurred at the center of the diamond search pattern, proceed to Step 3; if it did not so, proceed to Step 2.

**Step 2**

Keep the minimum block distortion finding from previous procedure on the center of LDSP. If the new minimum block distortion point was located at the center position, proceed to Step 3, otherwise, keep on repeating this step.

**Step 3**

Switch the search pattern from LDSP to SDSP. The minimum block distortion point found in this step would be the best matching block. And the candidate point that gives the best match would be chosen as the motion vector.

From above description, DS has some advantages. One is, in large motion content, getting the optimal minimum using fewer points than other mentioned method. The second is, in large motion content, the fewer points than those of mentioned method which were used for getting the optimal minimum. Third, DS is still faster than the traditional method of 4SS as to small motion content. Fourth, DS is highly sensitive in all directions in terms of the searching mode. Fifth, DS is harder to fall into the local minimum, as comprising with those mentioned methods. In contrast, the best point with the smallest distortion has more chance to locate nearest the smallest group distortion basically on the assuming. Then, there must exist some redundant point which has been calculated, especially in low resolution. Such distribution of search points in DS pattern is inefficient in finding possible candidates in the next step. For example, in Figure 5(b), according to the assumption, if point “9” is the minimum or the nearest to the minimum among the marked points, the distortion for point “4” with distance $\sqrt{2}$ should most likely be smaller than that for point “5” with distance 2. Therefore, in Figure 5(b), the point marked “9” would be not a good case for being a winner in the next step. The same case applies to the point marked “13” and the points marked “9” and “11” in Figure 5(c) which are also not good candidates for the searching in next step. The reason which causes such condition to result in disadvantages above mentioned is that the diamond shape is not approximately enough a circle, but it is just a 90° rotation square. Such condition makes Zhu et al. [5] propose a circle-shaped to search pattern with a uniform distribution of a minimum number of search points which is desirable to achieve the fastest search speed uniformly. The following will mention the comments on DS algorithm implementation. When the search pattern (LDSP or SDSP) is near or at the search window boundary, the searching procedure would be confined within the search window boundary. Naturally, the checking points outside the search window are truncated. For this reason, except drawing the searching window, restricting the searching range for the search pattern is necessary.

### 2.2. Hexagon-Based Search Pattern (HEXBS)

With curiosity what mechanism behind makes the DS pattern speed improvement up those square-shaped search pattern inspires Zhu et al. [5] investigated the research. After an extensive and intensive examination of search pattern, Zhu et al. proposed a hexagon-based search algorithm that can achieve substantial speed improvement up the DS algorithm with similar distortion performance. At the same time, HEXBS demonstrates significant speedup gain over the diamond-based search. And there are two drawbacks behind DS improved. One is that DS is searching with diamond shape, which is not approximately enough a circle but just 90° rotation of a square. Therefore, a circle-shaped search pattern with a uniform distribution of a minimum number of search points is desirable to achieve the fastest search speed uniformly. Practically, a more circle-approximated search pattern in the motion field is attainable in which a minimum number of search points are distributed uniformly. Each search point can be equally utilized with maximum efficiency, where the redundancy among search points should be removed maximally. The other is that the large diamond search pattern has some redundancy among the search pong in terms of distance between neighboring points, especially in the lower resolution search. And of course, such distribution of search points in DS pattern is inefficient in finding possible candidates in the next step.

**Figure 7:**
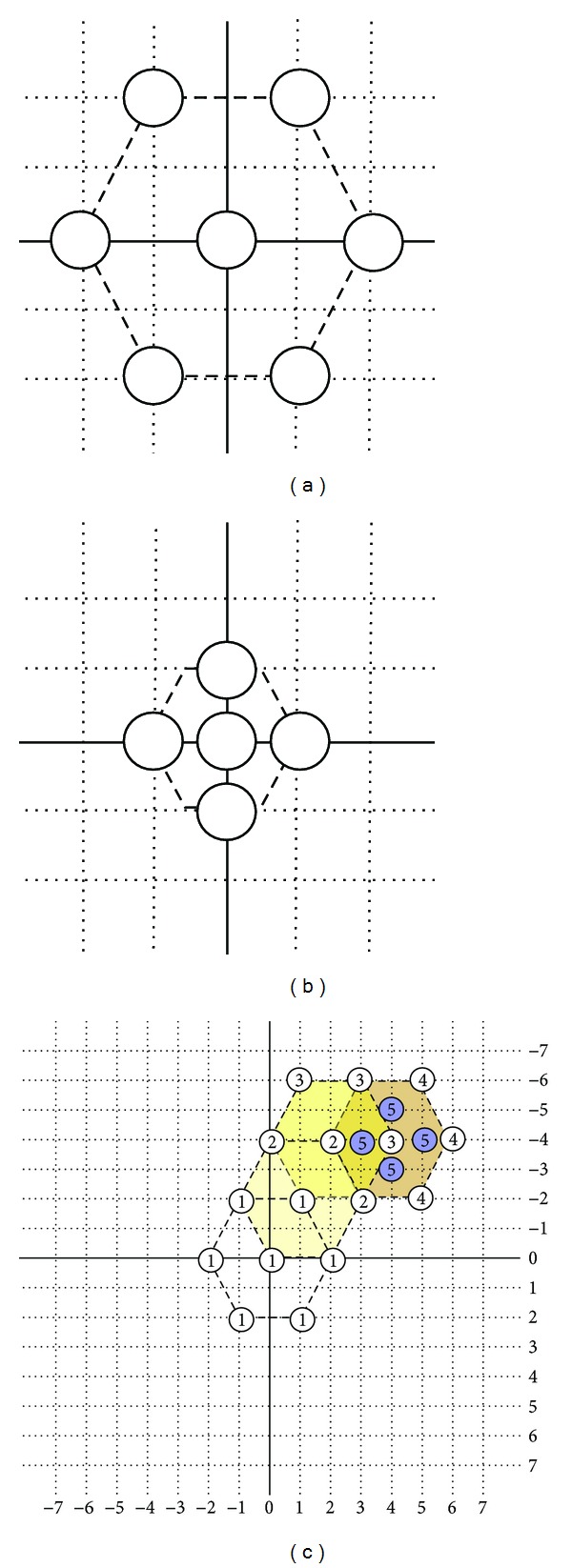
(a) Large HEXBS pattern. (b) Small HEXBS pattern. (c) Search path example.

A hexagon-based search pattern which is depicted in Figure 7(a) consists of seven checking points with the center surrounded by the six hexagon endpoints with the two edge horizontal points including up and down being excluded. Of the six endpoints in the hexagon, two horizontal points are away from the center point with distance of 2 and the other four points have a distance of $\sqrt{5}$ from the center point, respectively. That is, the distance between any neighboring pair of endpoints among the six endpoints is either 2 or $\sqrt{5}$. In other words, the six endpoints are approximately uniformly distributed around the center. On the other hand, the hexagonal search pattern also contains two fewer checking points than DS pattern with 9 points. In the search process, the hexagon-based search pattern keeps advancing with the center moving to any six directions. After calculating the first six endpoints of MSE, the minimum MSE will be obtained. No matter where the minimum MSE position is, three new endpoints will be produced with the overlapped three points which are centering the matching block distortion endpoint to form a new hexagon pattern. In the motion field, the focused inner search would be used finally. A smaller shrunk hexagonal pattern illustrated in Figure 7(b) covers four checking points around the center with distance of 1. The shrunken hexagonal pattern includes the same checking points the same as the small diamond search pattern. With the hexagonal search-point configuration, the search procedure is as follow. In the first step, the large hexagonal pattern with seven checking points is used for searching. If the optimum value was found at the center, the shrunken hexagonal pattern would be switched to be used for proceeding with four checking points of inner search, otherwise, the search continues around the point with minimum MSE using the same large hexagonal pattern. While the large hexagonal pattern moves along the direction of decreasing distortion, only three new nonoverlapped checking points will be evaluated as candidates each time.  Figure 7(c) shows an example of the search path strategy leading to the motion vector, where 20 search points are evaluated in five steps sequentially.

The first better candidate is (1, −2), obtained from the first large search pattern. Then, the next pattern will be centered (1, −2) producing three new endpoints to form another large search pattern. Candidates (2, −4) and (4, −4) are the smallest MSE for the third and the forth large HEXBS patterns, respectively. Consequently, the candidate (4, −4) is obtained for the final searching with the small HEXBS pattern. The HEXBS algorithm can be summarized in the following detailed steps.

**Step 1**

The original large hexagon with seven checking points is centered at (0,0), the center of a predefined search window in the motion field. If the best match point occurred at the center of the large hexagon, proceed to Step 3; if it did not so, proceed to Step 2.

**Step 2**

With the minimum MSE point in the previous search step as the center, a new large hexagon is formed with the three new candidate points and the nonoverlapped three old candidate points are checked. And the minimum MSE point is again identified. If the minimum MSE point was still the center point of the newly formed hexagon, then go to Step 3, otherwise, keep on repeating this step.

**Step 3**

Switch the search pattern from the large to the small size of the hexagon. The four points covered by the small hexagon are evaluated to compare with the current minimum MSE point. The new minimum MSE point is the final solution of the motion vector.

**Figure 8:**
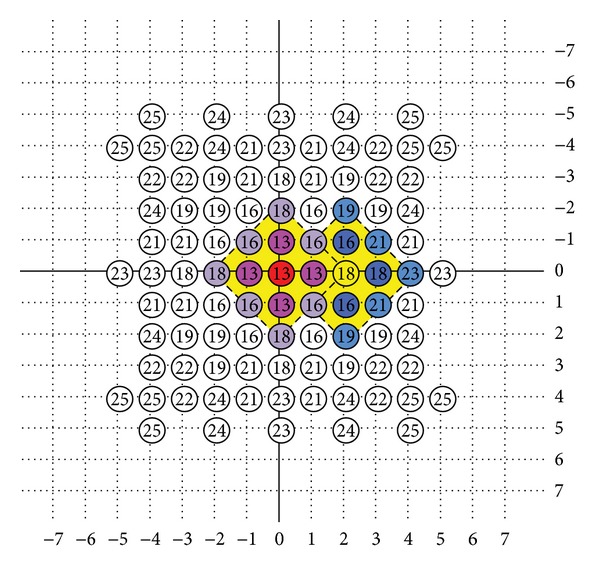
Minimum possible number of search points within the search window with HEXBS.

**Figure 9:**
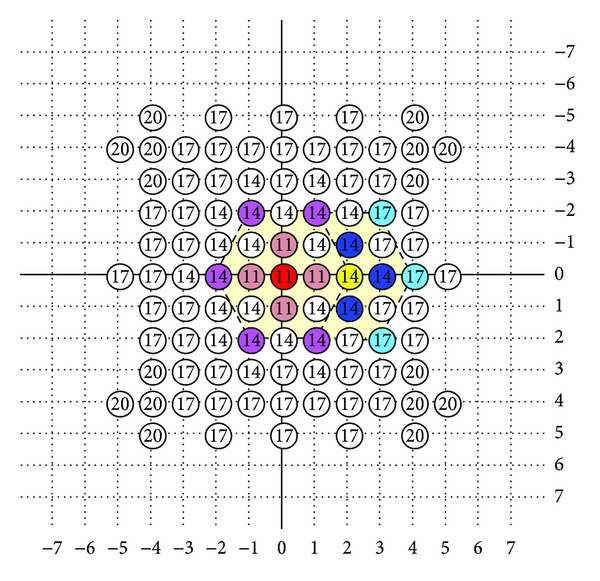
Minimum possible number of search points within the search window with DS.

**Figure 10:**
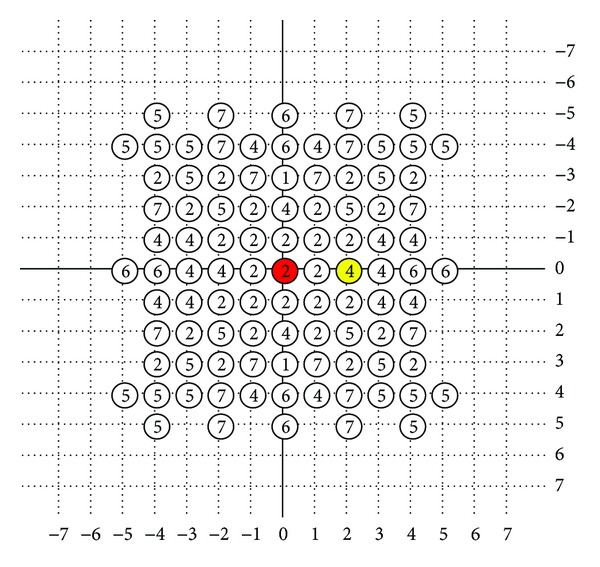
Number of search points saved by HEXBS compared with DS.

Here, the HEXBS algorithm will be examined to compare with DS algorithm in terms of number of points evaluated to find the same motion vectors. For the stationary motion vector (0,0), the DS algorithm checks 13 (= 9 + 4) blocks illustrated in Figure 8, whereas HEXBS algorithm evaluates 11 (= 7 + 4) blocks shown in the Figure 9. If (0, +2) was chosen, DS algorithm will need 18 block matches. However, HEXBS algorithm will need 14 search points. In such two conditions, two and four block matches can be saved using HEXBS algorithm as presented in Figure 10.

In short, the HEXBS scheme can find the same motion vector in the motion field with fewer search points than the DS algorithm. In Figure 10, can be seen that, it as the motion vector becomes farther from (0,0), more search points will be calculated. Generally speaking, the larger the motion vector is, the more search points the HEXBS algorithm can save. It is one of the reasons that causes the pattern of HEXBS algorithm to be used for designing the chromosomes for the proposed algorithm.

**Figure 11:**
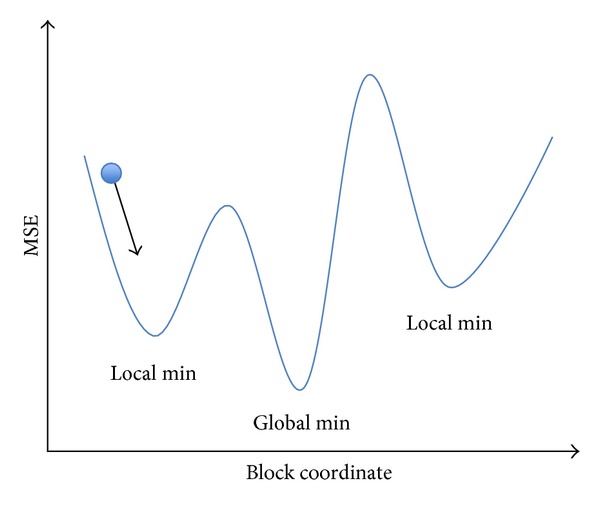
Fast block matching algorithm when getting stuck in the local minimum.

**Figure 12:**
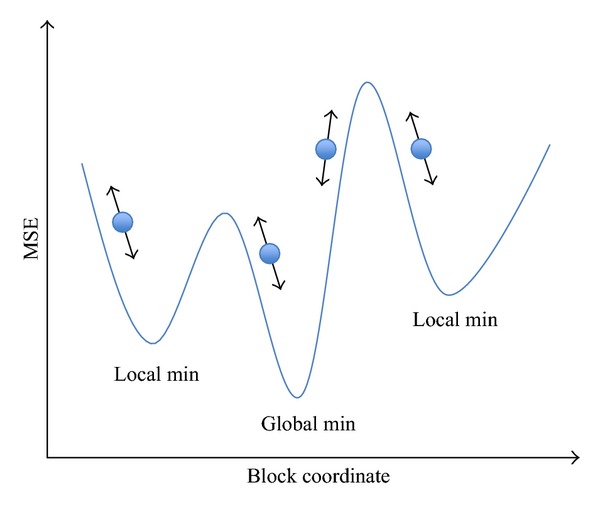
Block matching algorithm when there are more choices to get the global minimum.

The fast algorithms, which have been mentioned previously, are based on assumption that the distortion decreases monotonically as the search point gets closer to the global minimum. The assumptions cause the block matching falling into local minimum as illustrated in Figure 11. In the figure, the *x* coordinate is block candidates and the *y* coordinate is MSE. If the search path was always directed in the constant direction, the motion block matching would be easily gotten into local minimum. However, if the two characters of genetic algorithm and HEXBS algorithm were combined, the motion block matching would be not so easy to get into local minimum as shown in Figure 12.

**Figure 13:**
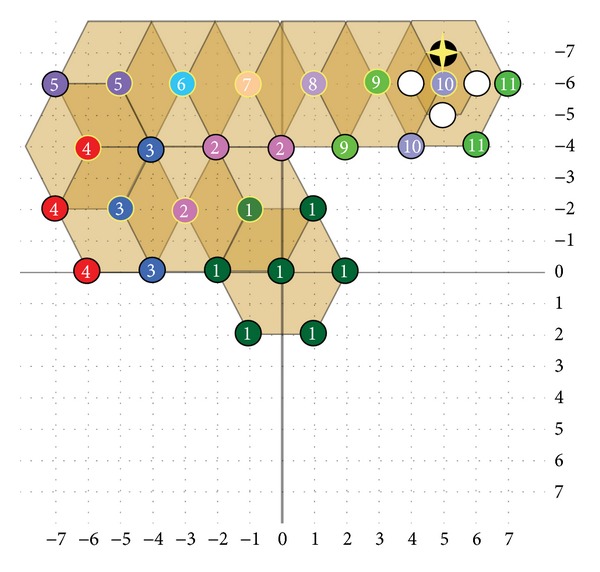
When getting the worse choice, the longer path would be processed.

After analysis of the research, many cases in a picture do not obey the assumption. On the contrary, they are usually falling into local optimal. On the other hand, after calculating, if there were two same minimum values, the process would have chance to get the longer way as shown in Figure 13. The coordinates (−1, −2) and (1, −2) have the same smallest value of MSE at the same time. If the moving direction was not going from the direction of (1, −2), it is of course will precede more points than choosing (1, −2) to calculate as illustrated in Figure 13. Besides, in this case, there are some points which will be never found in the searching procedure, such as (4, −5), (4, −7), (6, −5), and (6, −7).

The proposed algorithm will overcome these mentioned drawbacks. The proposed objective will be not only to get rid of the assumption to prevent the optimal value from falling into local minimum, but also to promote the search efficiency by combing the two techniques characters using genetic algorithm based on hexagon-based search pattern.

## 3. Genetic Algorithm for Hexagon-Based Motion Estimation

Genetic algorithm is one of the most familiar techniques of evolutionary computation. It was developed by John Holland over the course of the 1960s and 1970s and popularized by Goldberg. Genetic algorithm is a stochastic approach based on the concept of biological evolution and biological genetics. The operational process of genetic algorithm is based on the natural selection and natural genetics with the scheme that operate on chromosome-like data structures that encode possible solutions of the problem and apply crossover and mutation operations to generate new chromosomes in a search space. Genetic algorithm is capable of finding the near-optimal solution since the candidate solutions will not get stuck at the local optimal. Therefore, genetic algorithm is especially efficient when the search space of a problem has very rough search space riddled with many local optimal. Based on the principle of survival of the fittest, chromosomes with good performance are selected through selection operator.

**Figure 14:**
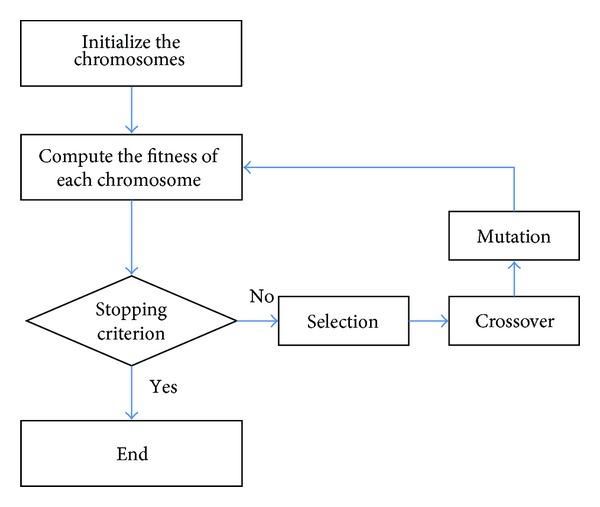
Genetic algorithm flow chart.

**Figure 15:**
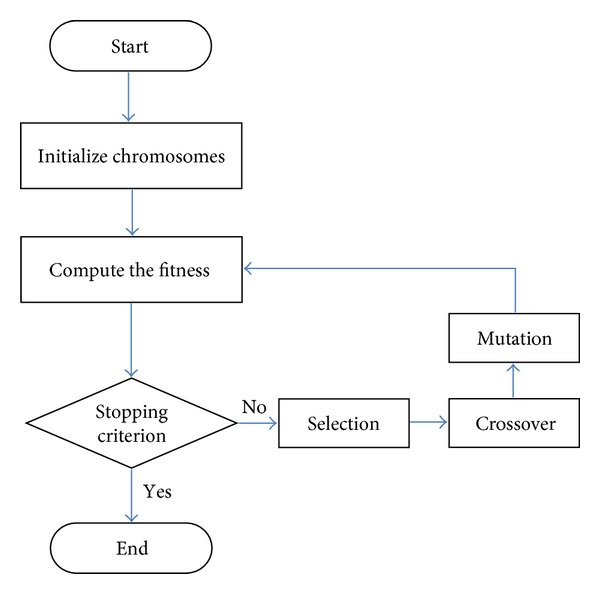
The proposed algorithm based on hexagon-based using genetic algorithm.

The flow chart of elementary operations of genetic algorithm is shown in Figure 14. First, both the chromosome formation and the fitness function are defined. The initial population is then randomly generated at the beginning of the evolutionary process. In each generation, the fitness values of all chromosomes in the current population are calculated and chromosomes with better fitness will be selected in the mating pool. Usually, two parents are selected from the mating pool for crossover in order to generate new offspring. The mutation operation is then applied to the offspring to maintain the diversity of the population and to avoid prematurity. Finally, a stopping criterion is tested to determine whether the evolution will go on or not. The proposed technology is based on hexagon-based search pattern using genetic algorithm. The flow chart is shown in Figure 15.

The detailed explanations of the terms of genetic algorithm are stated as follows.

**Step 1**

*Chromosome Formation:* first, one needs to encode the corresponding parameters of the problem to be solved, which is regarded as the kernel of the problem. Next, the parameters are coded as a finite length string called a chromosome. Usually, the chromosome can be denoted by real number or bit string depending on the problem under consideration. Here, the shortest path of the optimal matching block distortion based on HEXBS will be used. Some detailed definitions will be introduced in latter section.

**Step 2**

*Initial Population:* before genetic algorithm begins; a set of chromosomes should be created, usually randomly selected. This set of chromosomes is called the initial population. In the technique, there are six chromosomes which will be used. After experimenting on the random mode, the experimental results could not keep on the stable performance. In the technique, the initial population will choose the path of the previous optimal value with some specific blocks as illustrated in Figure 19 and the first HEXBS algorithm instead of random mode. From the experimental result, it is demonstrated that the method is better for using.

**Step 3**

*Fitness Function:* the fitness value measures how good a chromosome is. And the selections are made essentially according to this fitness value. In the proposed scheme, the fitness function is MSE^−1^.

**Step 4**

*Selection:* in the first generation, the goal of selection is to generate parents for the mating pool. Chromosomes of larger fitness values will be selected with higher probability. Common selective methods are random selection, fitness-proportionate selection that is also called roulette wheel selection, ranking selection, and tournament selection. After the first generation, the larger fitness values will be selected. In the paper, there are two of six worse chromosomes which will be given up. Then, the two chromosomes will be replaced by the two new chromosomes that are produced by crossover procedure.

**Step 5**

*Crossover:* in genetic algorithm, crossover operator makes the chromosome pool tend to convergence. Because the population size is not large in the proposed scheme, the chromosomes would be sorted and the best two of six chromosomes would be selected for crossover to replace the discarded two ones. That is, the crossover operation creates two new chromosomes from the best two existing chromosomes by exchanging some genes. After the procedure, the six chromosomes will be sorted again for choosing the worse two chromosomes for proceeding mutation.

**Step 6**

*Mutation:* the mutation process includes reproduction and survival competition. The mutation generates two new mutated chromosomes from the two worse chromosomes through mutation operators. Finally, the survival competition sorts and updates mutated chromosomes in terms of fitness values in a descending order. Mutation introduces new genes into chromosome, which are selected to perform mutation operator in order to prevent the chromosome pool from falling into a local optimum.

**Step 7**

*Stopping Criterion:* the iteration will be terminated in the matching generation. In the proposed scheme, in order to obtain the best performance, three, eight, fifteen, and twenty generations are used for testing. The experimental results demonstrate that, the increasing of the generation does not exactly increase the performance. For this reason, the proposed scheme GAHX with 3 generations for the following experimental results comparison is used.

**Figure 16:**
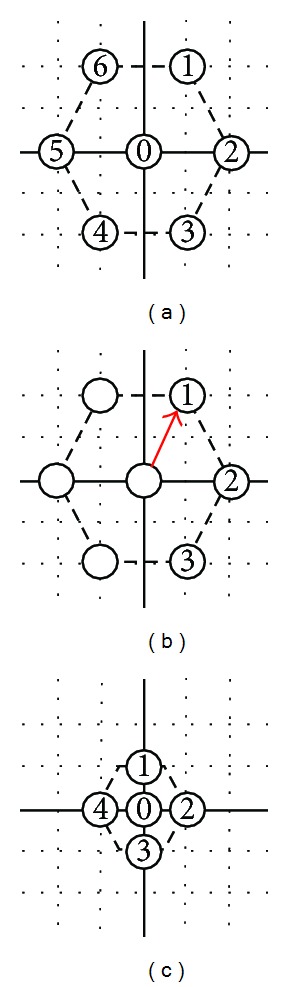
The definition of directions in gene on chromosome.

Here, the parameter definition of genes in the chromosome would be set first. The first gene, *G*_0_, in the chromosome has six direction candidates as shown in the Figure 16(a). If the direction is toward 1 o'clock, *G*_0_ will be set as one, and so forth. This is the first parameter for the first of five genes in the designed chromosome. As for the definition of the genes in the chromosome from *G*_1_ to *G*_3_, there would be three new direction candidates. The idea is based on HEXBS. When moving, three new endpoints would be created. That is to say those three new paths will be created for candidates. The first one direction obeys the clockwise as shown in Figure 16(b) will be set as one. Finally, *G*_4_ has four direction candidates. The first one would be marked on the direction of twelve o'clock as shown in Figure 16(c).

**Figure 17:**
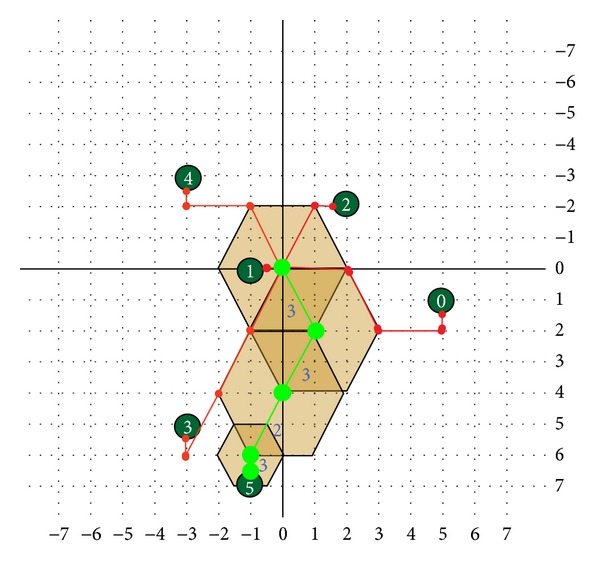
Some examples of selecting chromosomes.

**Figure 18:**
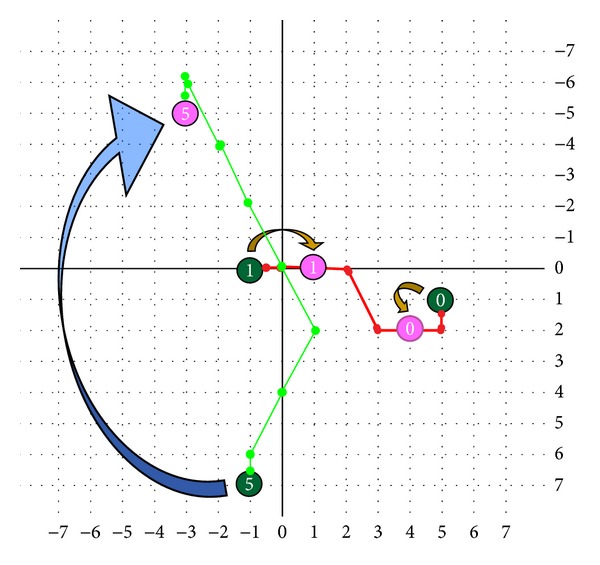
Some examples of chromosome, crossover, and mutation.

In Figures 17 and 18, some examples about chromosomes, crossover, and mutation are shown. If one of the chromosomes is {3,3,2,0,3}, the first step would go from (0,0) to (1,2) and then get the position (−1, 6) through (0,4), and finally the point (−1, 7) would be obtained as depicted in Figure 17. In Figure 17, the two chromosomes are {2,3,2,0,1} and {0,X,X,X,4}. After crossover, the two chromosomes would become {2,3,2,0,4} and {0,X,X,X,1}. The position would be from (−1, 0) to (1,0) and from (5,1) to (4,2) as illustrated in Figure 18. That is, crossover would just swap the gene {*G*_6_} of the two chromosomes.

In contrast, the mutation would be just limited to mutate the gene *G*_0_. The example is shown in Figure 18. The chromosome will be mutated *G*_0_, from {3,3,2,0,3} changing to {6,2,2,0,3}, that is to say the mutation is changing the gene from 3 to 6. The position of the chromosome will be changed from (−1, 7) to (−3, −5).

**Figure 19:**
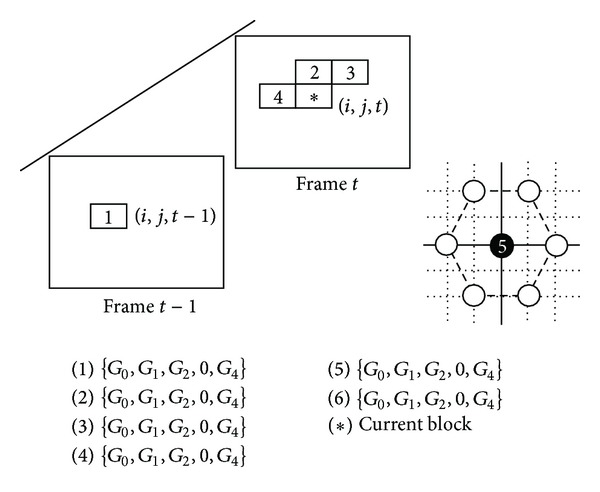
The initial six chromosomes selection.

In our early experiment, the performance using the proposed scheme could not perform very well in some video sequence all the time. After the analyzing the algorithm, the selection chromosomes in Genetic Algorithm is using random mode without any obeying rules. It is the reason which causes the performance could not bring the proposed technique into full play. Expecting to obtain a better efficiency, the candidate chromosomes are the focused problem to overcome. For the characters of image spatial and temporal [14], the initial four chromosomes selection are succeed to the best motion vector with blocks marked as 2, 3, and 4 in the current frame and the block mark as 1 which is the same block position in referenced frame as depicted in Figure 19.

By this way four chromosomes have been finished selecting. Besides, in order to take the advantages of HEXBS, the best point computed by the first seven blocks matching distortion of HEXBS will be regarded as one of the six chromosomes. In other words, the five chromosomes selections have been finished. The last one chromosome will be selected from random mechanics. The selected six chromosomes are the initial chromosomes for the proposed GAHX. The performance for the promoting results has been presented in the following experimental results.

## 4. Experimental Results

In order to compare the performance of different search algorithms, full Search, hexagon-based search pattern (HEXBS), and hexagon-based motion estimation using genetic algorithm (GAHX) are implemented in the following simulations. The experimental results will be presented in this section. In the simulation, the distortion measurement of mean square error would be used with block size of 8 × 8 and search window size of 15 × 15. Six standard video sequences were used. They are “Football” (90 frames), “Forman” (400 frames), “Bus” (150 frames), “Stefan” (300 frames), “Mobile” (300 frames), and “Erik” (50 frames), with the size of 352 × 288. In the proposed scheme, these video sequences are classified into three kinds of motion content: objects in video sequence are changing very violently, the moving of objects in video sequence is slow, and the background is almost static, respectively.

**Figure 20:**
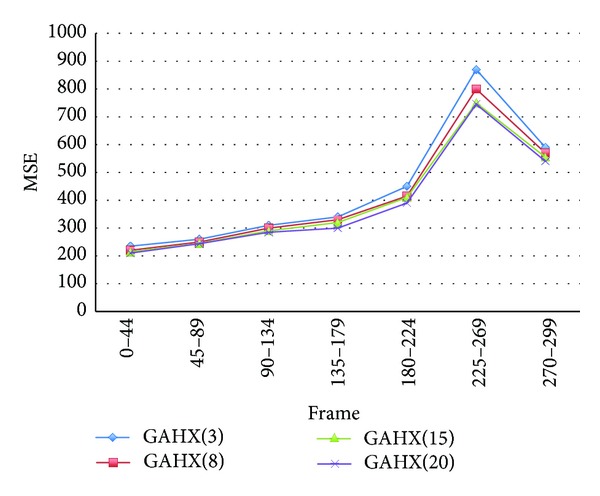
Average MSE per block for Stefan video sequence using various generation with the proposed technique.

**Figure 21:**
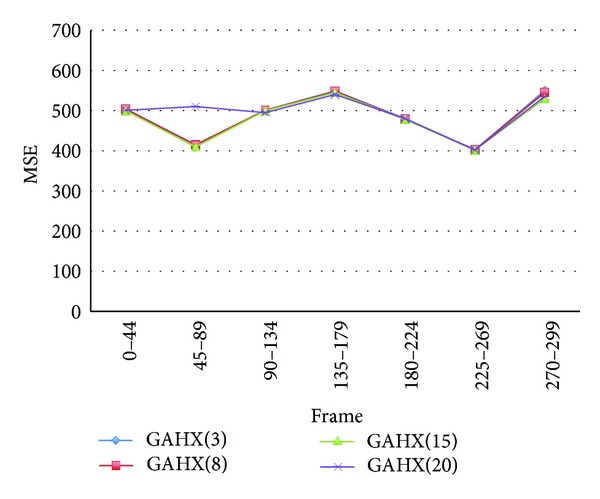
Average MSE per block for Mobile video sequence using various generations with the proposed technique.

The average mean square error values, average percent rate per frame of PSNR worse than 30 dB, and search point numbers are summarized as in figures and tables for various algorithms, including full search, HEXBS, and the proposed algorithms GAHX, respectively. To obtain the best performance, the tests for the proposed GAHX with three, eight, fifteen, and twenty generations are performed. The results compared with various video sequences using the proposed scheme are depicted in Figures 20 and 21. These figures demonstrate that the increasing of the generation does not exactly increase the performance. This can be easily seen in Figure 20, especially between the 225th and 269th frames of the Stefan video sequence. Although the average mean square error per block is decreasing from three to twenty generations, the decreasing from fifteen to twenty generations is not very obvious.

For this reason, the proposed scheme GAHX with three generations for the following experimental results comparison is used.  Table 1 is the average mean square error per block in full search, HEXBS, and GAHX with three generations compared with various video sequences classified into three kinds of conditions. From Table 1, it can be seen that average mean square error per block using GAHX with three generations is better than the technique of HEXBS for Bus and Stefan video sequences. In the Bus video sequence, the average mean square error is 122.0, 336.3, and 256.2 with full search, HEXBS, and GAHX perform three generations, respectively.

Although the mean square error values of Football, Forman, and Erik video sequences are not superior to HEXBS, the difference of average mean square error compared to HEXBS is just between 1.3 and 8.2. One thing worth to mention is that the average mean square error per block of the proposed GAHX scheme with three generations even is not superior to HEXBS in Football video sequence. In football video sequence, the average worse percent rate per frame of PSNR is decreased almost the same as full search between the 18th and 35th frames. After analyzing, the reason causing such high performance is that the content in the section of Football video sequence is from many objects of human transfers to only one human and a passing football with grassland.

From Table 1, it can be easily obvious that the changing of objects in whole sequence is very violent. The average mean square error per block of the proposed scheme with three generations would be superior to HEXBS. And the difference of average mean square error per block between HEXBS and GAHX(3) can reach 80.1. Using the proposed GAHX(20) in Bus video sequence, the difference of average mean square error per block can reach 96.8 as depicted in Table 1 when comparing to HEXBS technique. In other words, the proposed method performed highly especially when the objects in video sequence change violently.

**Figure 22:**
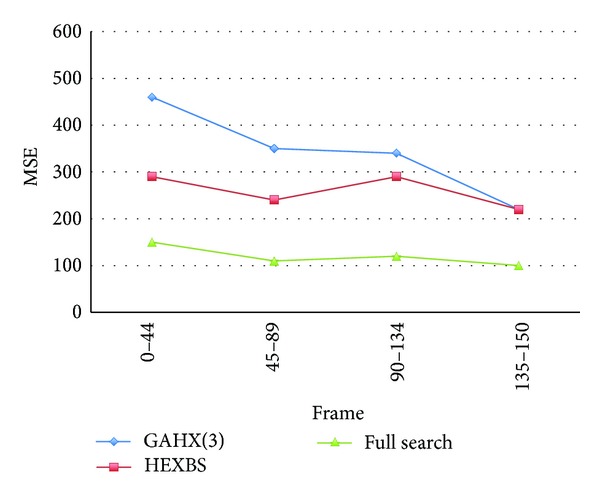
Average MSE per block compared with different methods for Bus video sequence.

**Figure 23:**
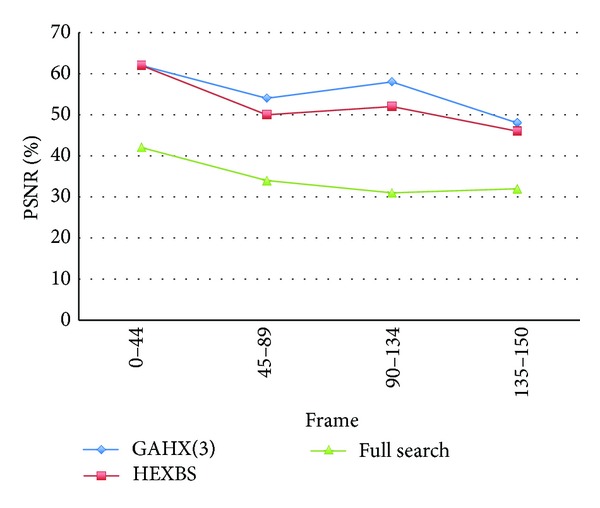
Average percent rate per frame of PSNR worse than 30 dB compared with various schemes for Bus video sequence.

In Figures 22 and 23, the diagrams of the curves performed by the proposed scheme are always smaller than those performed by HEXBS. For the kind of video sequence such as Bus video sequence, the performance is promoted especially better than the same video sequence using HEXBS.

**Figure 24:**
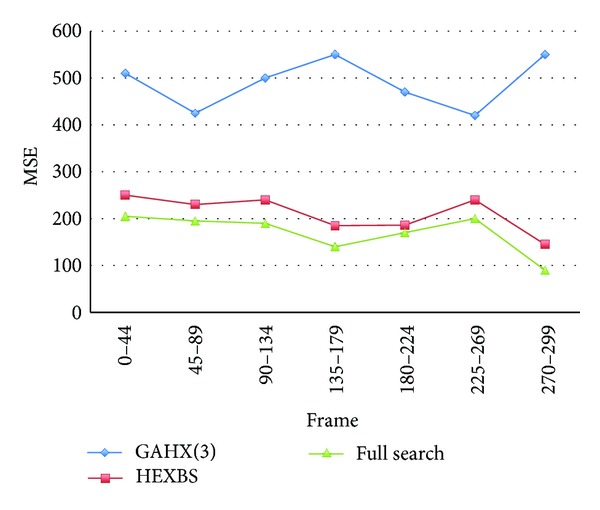
Average MSE per block compared with different methods for Mobile video sequence.

**Figure 25:**
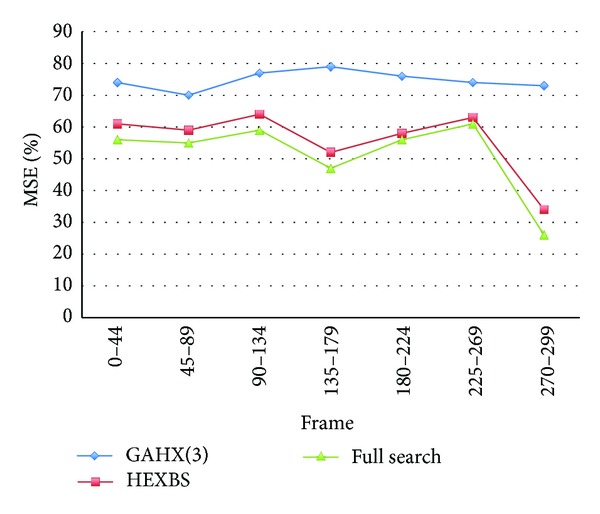
Average percent rate per frame of PSNR worse than 30 dB compared with various schemes for Mobile video sequence.

In contrast, the changing of Mobile video sequence with objects in video content is not violent; most of the motion vectors can be obtained near the original position. The experimental results have verified the statement that, with the Mobile video sequence, using GAHX is a worst case as shown in Tables 1 and 2 and Figures 24 and 25. We could be easily to seen that the values and the diagrams of the curves performed by the proposed technique are always far from those by HEXBS. So it is not suitable to use GAHX in such kinds of video sequences.

## 5. Conclusions

HEXBS speeds up searching points the most among the other fast search methods. According to our experimental results, HEXBS is easy to get into local minimum as the object in video sequence change violently. In order to overcome this drawback, the proposed algorithm integrates the characteristics of HEXBS and genetic algorithm. The experimental results have demonstrated higher performance in video sequence with objects changing violently. In contrast, the proposed technique GAHX does not perform well for objects in video content changing slowly.

## Figures and Tables

**Table 1:** Average MSE per block in various methods with different video sequences.

Classify	High activity			Medium activity		Low activity
Video sequence	Football	Bus	Stefan	Forman	Erik	Mobile

FS	150.6	122.0	336.5	44.3	31.0	170.0
HEXBS	222.6	336.3	444.7	68.0	49.4	207.8
GAHX(20)	190.6	239.5	389.3	83.0	59.2	485.9
GAHX(15)	194.9	238.5	396.2	74.7	54.1	485.7
GAHX(8)	206.0	247.6	408.2	73.6	56.0	485.4
GAHX(3)	223.9	256.2	423.8	73.6	57.6	488.8

**Table 2:** Average percent rate of PSNR worse than 30 dB per frame for various video sequences compared with various schemes.

Video sequence	Football	Bus	Stefan	Forman	Erik	Mobile
FS	29%	34%	51%	15%	12%	50%
HEXBS	36%	56%	53%	22%	16%	56%
GAHX(20)	33%	52%	56%	27%	17%	74%
GAHX(15)	33%	52%	56%	25%	17%	74%
GAHX(8)	34%	52%	56%	24%	17%	74%
GAHX(3)	35%	53%	56%	24%	17%	74%
